# Germline Genetic Testing in Patients with Bone and Soft Tissue Sarcoma: A Prospective Multicenter Study to Evaluate Cancer Susceptibility †

**DOI:** 10.3390/ijms26072839

**Published:** 2025-03-21

**Authors:** Isaak Ailts, Michael A. Golafshar, Katie L. Kunze, Margaret Klint, Kathleen Barrus, Robert L. Nussbaum, Edward D. Esplin, Brandie Leach, Sarah Young, N. Jewel Samadder, Mahesh Seetharam

**Affiliations:** 1 Division of Hematology and Medical Oncology, Department of Medicine, Mayo Clinic, Phoenix, AZ 85054, USA; isaak.ailts@und.edu; 2 Division of Hematology/Oncology, University of North Dakota, Fargo, ND 58122, USA; 3 Department of Quantitative Health Sciences, Mayo Clinic, Phoenix, AZ 85043, USA; 4 Department of Clinical Genomics, Mayo Clinic, Phoenix, AZ 85043, USA; 5 Labcorp Genetics (formerly Invitae), San Francisco, CA 94103, USA; 6 Division of Gastroenterology & Hepatology, Department of Medicine, Mayo Clinic, Phoenix, AZ 85054, USA; 7 Center for Individualized Medicine, Mayo Clinic, Phoenix, AZ 85054, USA

**Keywords:** sarcoma, germline testing, homologous recombination deficiency, genetic testing

## Abstract

Sarcomas are rare heterogenous mesenchymal tumors with over seventy-five different subtypes, with varying biology and outcomes, with no clear inciting factor in the vast majority. To determine the prevalence of pathogenic germline variants (PGV) in patients with sarcomas, we undertook a prospective multi-site study of germline sequencing using an 84-gene next-generation sequencing panel among patients receiving care at the four Mayo Clinic Cancer Centers. Of 115 patients with bone and soft tissue sarcoma, the median age was 60 years, 49.6% were female, 82.6% were White. The anatomical location of the primary tumor included extremities (34.8%), retroperitoneum (19.1%), trunk (13.0%), and head and neck (7.8%). Family history of cancer was present in 62.6% of the study population. Ten patients (8.7%) had a pathogenic/likely pathogenic variant (PGV). Of these, three had stage IV sarcoma, and seven had earlier-stage sarcoma (stages I–III). Among the 55 (48.7%) patients who had variant of uncertain significance (VUS), 41.1% (22/55) had stage IV sarcoma and 58.9% (33/55) had earlier-stage disease. Of the ten patients with PGV, high-to-moderate penetrance gene abnormalities were identified in eight patients (80%) involving *TP53* (3), *BRCA1* (1), *SDHA* (1), *ATM* (2), and *NBN* (1) genes. The vast majority of the PGVs (70%) would not have been detected using the current guidelines. Because of the paucity of sarcomas and lack of effective treatment options for advanced disease, germline testing in sarcomas represents a potentially impactful strategy to assess therapeutic options and for assessment of familial risk.

## 1. Introduction

Sarcomas are a heterogeneous group of tumors stemming from mesenchymal tissue. They comprise approximately 1% of adult yet 15% of child malignancies, lending credence to a possible genetic predisposition to sarcomas [1]. The annual incidence of soft tissue sarcoma is about 15,000–16,000/yr. in the United States [2]. Sarcomas are very heterogenous, with over seventy-five different histologic subtypes which have different clinical and histological characteristics [3]. There are known, well-established, genetic cancer syndromes that involve sarcoma including in Li–Fraumeni syndrome (*TP53*) and Carney–Stratakis syndrome (*SDHB*, *SDHC*, *SDHD*), among other syndromes [4,5,6]. The mainstay of sarcoma treatment is surgical resection and radiotherapy with approved systemic therapies including chemotherapy and targeted treatments, which have shown improvement in progression-free survival and, in some instances, overall survival.

Research in genomics continues to discover new genetic variants that may increase risk for malignancies, with a recent study detecting up to 8% of pathogenetic/likely pathogenic germline variants (PGV) across over 10,000 cancer cases, including novel gene/malignancy associated risk [7]. Other studies have demonstrated this trend specifically with sarcomas in several adult cohorts, supporting the idea that sarcomas may have genetic predisposition that may be clinically significant yet underrecognized [8]. The National Comprehensive Cancer Network (NCCN) guidelines currently recommend genetic risk assessments in sarcoma if patients fall into syndromic specific scenarios. For example, in Li–Fraumeni syndrome, NCCN recommends genetic testing in individuals with a diagnosis of sarcoma at ages younger than 45 years of age and with a first-degree relative diagnosed under the age of 45 with any kind of cancer and a first- or second-degree relative with sarcoma at any age and an additional cancer under 45 years [9].

Targeted precision therapies are available for multiple cancer types including sarcomas in second line and beyond with modest efficacy [10]. A recent cross-sectional study has shown that slightly less than half of sarcoma patients had at least one potentially clinically relevant genetic alteration in the primary tumor [11]. Studies with molecularly predicated therapies have yielded favorable responses to targeted treatments such as Trabectedin in relapsed sarcoma patients with favorable BRCA1 haplotypes [12].

Germline genetic testing in unselected sarcoma patients is an underexplored realm. This paper aims to determine the prevalence of PGV in sarcomas using a universal genetic testing approach and compare to current genetic testing recommendations. Expanded testing has the potential to better understand familial genetic risk for sarcomas as well as other tumors and lead to us of more effective targeted therapies. In this article, we report the findings of a multicenter prospective cohort of soft tissue sarcoma patients who had multigene germline testing completed.

## 2. Results

**Figure 1:**
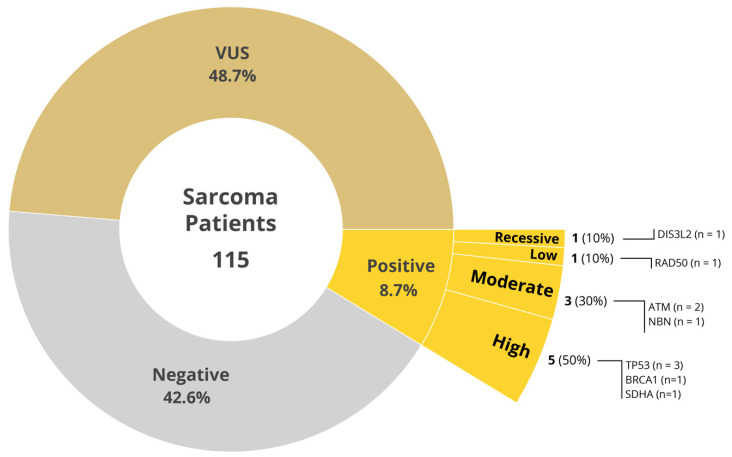
Distribution of germline testing results.

Demographic characteristics of the study population are shown in Table 1 and stratified by results of variant germline analysis. Of the 115 sarcoma patients evaluated, 10 patients (8.7%) had a PGV including 8 in high and moderate penetrance genes (Figure 1). No PGVs were observed in 49 patients (42.6%) while VUS were found in 56 patients (48.7%). Overall, 82.6% of patients were white with females comprising about half of patients (49.6%) across the three sites. Median age was 60.0 across the study without significant difference across PGV groups. Additional demographic risk factors across the population included smoking history (32.2%), BMI > 30 (31.3%), diabetes mellitus (12.2%), and hypertension (46.1%), none of which were meaningfully different among PGV groups.

The anatomical location of primary tumor included extremities (34.8%), retroperitoneum (19.1%), trunk (13.0%), head and neck (7.8%), as well as some uncommon areas including breast, heart, gastrointestinal tract, endometrium, inferior vena cava, liver, spleen, lungs, ovaries, vagina, bladder, and rectum. Over 32.7% of patients were metastatic, with PGV being metastatic 30% at the time of recruitment compared to 26.5% and 33.9% of patients with negative pathogenic variants and VUS, respectively. No formal statistical analyses were performed because of the small group size for the positive cohort.

Family history of cancer was present in 62.6% of the study population. Pedigrees were completed in fifty-eight of the study participants with a first-degree relative with any cancer occurring in thirty-seven patients (32.2%). Family history of cancer was determined based on a combination of the pedigree and information found in the patient’s electronic health record. Of the ten patients with PGV, high to moderate penetrance genes comprised 80% as seen in Figure 1. Of these variants, deletions were observed in 6 of the 10 (60%), while missense mutations were present in the remaining 4 (40%). When applying 2020 National Comprehensive Cancer Network (NCCN), National Society of Genetic Counselors (NSGC), or American College of Medical Genetics and Genomics (ACMG) guidelines for genetic testing, 70% of PGV would not have been detected by current guidelines for their primary cancer (Table 2). All three patients with TP53 mutation PGV met guidelines for testing in our study but only two out of three met Chompret guidelines for testing based on family history, and only one of three met NSGC/ACMG criteria for testing (2020 guidelines). Subsequent testing of family members of patients with pathogenic variants is being undertaken.

## 3. Discussion

Universal germline genetic testing in sarcoma patients led to the identification of 8.7% of PGV with high or moderate penetrance genes including *BRCA1*, *SDHA*, *TP53*, *ATM*, *NBN*, *RAD50*, and *DIS3L2* (Figure 1). Several of these genes have targeted treatment trials that could potentially be an effective option. This includes BRCA1 mutated tumors treated with trabectedin [12]. Clinical trials targeting variants in the *TP53* gene are also being under investigation [13]. These detected variants may represent additional opportunities for precision medicine targeted therapies for the patient as well as their family members who may be at risk of developing similar familial neoplasms.

One of the significant findings of our study lies in the fact that 70% of PGV would have been missed by current recommendations for germline testing utilizing 2020 guidelines. There are currently no specific testing germline testing guidelines for most sarcomas unless they fall into the category of hereditary tumor predisposition syndromes, such as Li–Fraumeni syndrome, which require a detailed and expansive family history of other malignancies. These guidelines are currently quite complicated and at times hard to apply clinically, as outlined in Table 3. The complexity of these guidelines makes their usefulness and applicability in busy oncology practices limited and, thus, deny necessary genomic testing to patients with sarcomas.

The current guidelines are extremely specific and limited in applicability leading to potential missed identification of PGV even if guidelines are adhered to, as shown in our study with 70% of PGV being missed if solely relying on current guidelines to inform genetic testing/referral. This could potentially lead to missed treatment opportunities including potentially beneficial clinical trials for the patient. It also could impact earlier screening or identification of family member at elevated risk leading to an earlier detection of cancer when resection would have led to more favorable outcomes. Four patients with PGVs had potential therapeutic options with either PARP inhibitors (BRCA and ATM mutations) or clinical trials targeting the HRD pathway for RAD50 mutation. One of the two patients with ATM mutation briefly received Olaparib but she progressed within 3 months, and insurance denied approval of the drug for the second patient. The patient with BRCA mutation was treated with Olaparib and remained progression free for 4.5 months. The patient with RAD 50 mutation was evaluated for a clinical trial targeting HRD pathway, but due to suboptimal performance status was ineligible. The 32 y/o patient with SDH mutation GIST was referred to another sarcoma center for clinical trial with Temozolomide. This highlights the importance of testing earlier in the disease course, as there is a higher possibility of patients’ condition allowing for enrollment in clinical trials and allows time for insurance approval process, which is important based on our experience with our patient when it took longer to go through the process as it was considered off-label use. Identification of VUS on multigene panel testing is almost inevitable, and previous reports have shown the expected frequency of VUS correlates with panel size [15]. We report a VUS rate of 48.7%, which is consistent with rates observed in prior studies involving multigene panel testing of patients with cancer [7,16]. Post-test referral of patients with VUS results to a genetic counselor or clinical geneticist is an effective approach to help further mitigate these concerns.

Our study highlights the importance of genomic testing in sarcoma patients, as it might uncover clinically actionable alterations and potentially impactful variants of uncertain significance, which might add to the scientific literature for future reference. In recent years, there has been multiple published articles focused on germline testing of patients with various cancers including sarcoma. These have shown pathogenic variants in some of the known reported alterations including TP53, ATM, BRCA2, and ERCC2 [17], and in some of the emerging variants including PALB2, RAD50, and FANCM [16]. Another study in Middle Eastern patients showed a 20.7% incidence of PGVs, which correlated with younger age of cancer diagnosis, a second primary, and female gender with potentially actionable alterations [17]. Our study, in addition to known PGVs in sarcomas, also showed two unusual PGVs that are not, including DIS3L2 deletion and NBN deletion (Table 4). The DIS3L2 gene is reported to have a critical role in RNA metabolism and is proposed as an attractive target for novel therapeutics [18]. The NBN gene is reported to be a contributor to a broad cancer spectrum [19] and may have contributed to the multiple cancers in our patient’s family. There are no currently approved therapies for these two uncommon genomic variants.

Limitations of this study include demographics across the multiple Mayo sites that may not represent other areas of the United States or other countries due to demographic differences represented in the study. This study also contained significant diversity in the types of sarcomas with a limited number of specific subtypes of sarcoma, which, while representative of the sarcoma populations, makes it difficult to draw specific conclusions of individual sarcoma subtypes. Comparison of germline and somatic mutations was not performed for this analysis. Additional large cohort studies will help guide appropriate guidelines recommendations. Further studies could include addition of clinical outcome data compared by PGV to better understand response to therapies and correlation to somatic mutation results.

## 4. Methods and Materials

### 4.1. Patient Recruitment

Following approval from the Mayo Clinic Institutional Review Board (18-000326), across the Mayo Clinic Enterprise at four Cancer Center sites (Phoenix, AZ; Jacksonville FL; Rochester, MN; and Eau Claire, WI), a total of 2984 patients with active solid tumor malignancies aged 18–85 with various cancers were recruited via written informed consent from 1 April 2018 through 31 March 2020 in the Interrogating Cancer Etiology Using Proactive Genetic Testing (INTERCEPT) Program [20]. The study was limited to patients older than 19 years due to predominance of soft tissue sarcoma in adults, and non-availability of pediatric oncology service at all sites. Prior to participation, patients viewed a pretest education video and provided with pretest genetic counseling upon request. Research coordinators recruited patients in clinic for treatment across sites from radiation oncology, medical oncology, surgical oncology, and dermatology clinics. Age, ethnicity, family history of cancer, stage at diagnosis, multifocality, or prior malignancy were not exclusionary. Descriptions of the full cohort of patients in the INTERCEPT study have been published [20]. All patients underwent the Invitae Multicancer next-generation sequencing (NGS) panel of 83 genes (84 beginning July 2019) at no cost. Patients with pathogenic variants were provided with individual genetic counseling to discuss the potential impact of their variant on their treatment. Family cascade testing was offered to first-degree relatives of patients with PGVs at no additional cost within 90 days.

### 4.2. Data Collection

Patient demographics, disease characteristics, treatment information, clinical outcomes, and family history were collected by medical record review or questionnaires administered directly to patients. Analyzed data were deidentified except to study investigators and stored in a secure database. The 115 sarcoma patients with a diagnosis of sarcoma were recruited to the INTERCEPT study and included in the current analysis. No patients were enrolled at the Eau Claire, WI site.

### 4.3. Genetic Sequencing

Genetic sequencing and interpretation of genetic findings were performed by Labcorp Genetics (formerly Invitae, San Francisco, CA, USA) as described previously. All variant findings were independently verified by a medical geneticist. Sequencing results were categorized for PGVs as high, intermediate, or low penetrance; recessive; or as a variant of uncertain significance (VUS).

### 4.4. Statistical Analysis

We used descriptive statistics to explore patient demographics, disease characteristics, treatment information, and clinical outcomes in this cohort. Results of the genetic sequencing with rates of PGVs and VUS/negative results were determined. Rates of incremental findings for those who would have been missed under current testing guidelines for sarcoma were calculated. Tables and figures were developed using R version 4.2.2. [21]

## 5. Conclusions

This prospective, multi-site study, comprising over one hundred patients with diverse sarcomas, using universal unselected germline testing, identified PGV in 8.7% of patients, with a majority of these expected to have been missed by current targeted germline genetic testing guidelines. Overall, given the diversity and paucity of sarcomas, combined with their limited advanced disease treatment options, universal germline testing in sarcomas represents an interesting and potentially impactful strategy to better characterize potential genetic underpinning in sarcomas, understand familial risk allowing for early-stage detection in at-risk patients, and the potential to offer targeted precision cancer treatments to improve outcomes.

## Figures and Tables

**Table 1:** Clinical and demographic characteristics of included patients.

	All	Positive	Negative	VUS
**N**	115	10	49	56
**Enrollment site**				
Midwest	32 (27.8%)	3 (30.0%)	11 (22.4%)	18 (32.1%)
Southeast	29 (25.2%)	3 (30.0%)	14 (28.6%)	12 (21.4%)
Southwest	54 (47.0%)	4 (40.0%)	24 (49.0%)	26 (46.4%)
**Gender**				
Male	58 (50.4%)	4 (40.0%)	33 (67.3%)	21 (37.5%)
Female	57 (49.6%)	6 (60.0%)	16 (32.7%)	35 (62.5%)
**Age**				
Mean (SD)	56.6 (15.8)	48.3 (15.9)	60.1 (16.2)	55.0 (14.9)
Median	60.0	47.0	65.0	56.5
Range	19.0–79.0	26.0–73.0	20.0–79.0	19.0–79.0
**Race (grouped)**				
White	95 (82.6%)	7 (70.0%)	41 (83.7%)	47 (83.9%)
Hispanic/Latino	5 (4.3%)	1 (10.0%)	2 (4.1%)	2 (3.6%)
Black/African American	4 (3.5%)	1 (10.0%)	1 (2.0%)	2 (3.6%)
Asian	2 (1.7%)	0 (0.0%)	1 (2.0%)	1 (1.8%)
American Indian/Alaskan Native	3 (2.6%)	0 (0.0%)	1 (2.0%)	2 (3.6%)
Native Hawaiian/Pacific Islander	0 (0.0%)	0 (0.0%)	0 (0.0%)	0 (0.0%)
Other	6 (5.2%)	1 (10.0%)	3 (6.1%)	2 (3.6%)
**History of Smoking**				
Yes	37 (32.2%)	3 (30.0%)	18 (36.7%)	16 (28.6%)
No	78 (67.8%)	7 (70.0%)	31 (63.3%)	40 (71.4%)
**Body Mass Index >30**				
Yes	36 (31.3%)	4 (40.0%)	11 (22.4%)	21 (37.5%)
No	79 (68.7%)	6 (60.0%)	38 (77.6%)	35 (62.5%)
**Diabetes Mellitus**				
Yes	14 (12.2%)	3 (30.0%)	6 (12.2%)	5 (8.9%)
No	101 (87.8%)	7 (70.0%)	43 (87.8%)	51 (91.1%)
**Hypertension**				
Yes	53 (46.1%)	3 (30.0%)	23 (46.9%)	27 (48.2%)
No	62 (53.9%)	7 (70.0%)	26 (53.1%)	29 (51.8%)
**Prior History of Radiation**				
Yes	5 (4.3%)	2 (20.0%)	0 (0.0%)	3 (5.4%)
No	110 (95.7%)	8 (80.0%)	49 (100.0%)	53 (94.6%)
**Family History of Cancer**				
Yes	72 (62.6%)	7 (70.0%)	31 (63.3%)	34 (60.7%)
No/unknown	43 (37.4%)	3 (30.0%)	18 (36.7%)	22 (39.3%)
**Pedigree Complete**				
Yes	58 (50.4%)	5 (50.0%)	31 (63.3%)	22 (39.3%)
No	57 (49.6%)	5 (50.0%)	18 (36.7%)	34 (60.7%)
**Location**				
Extremities (upper/lower)	40 (34.8%)	2 (20.0%)	14 (28.6%)	24 (42.9%)
Retroperitoneum	22 (19.1%)	1 (10.0%)	8 (16.3%)	13 (23.2%)
Trunk	15 (13.0%)	2 (20.0%)	8 (16.3%)	5 (8.9%)
Head and neck	9 (7.8%)	1 (10.0%)	4 (8.2%)	4 (7.1%)
Other	29 (25.2%)	4 (40.0%)	15 (30.6%)	10 (17.9%)
**Histology**				
Mixed types (carcinosarcoma)	1 (0.9%)	0 (0.0%)	0 (0.0%)	1 (1.8%)
Sarcoma	114 (99.1%)	10 (100.0%)	49 (100.0%)	55 (98.2%)
**Histology Subgroup**				
Liposarcoma	17 (14.8%)	1 (10.0%)	8 (16.3%)	8 (14.3%)
Leiomyosarcoma	20 (17.4%)	1 (10.0%)	8 (16.3%)	11 (19.6%)
Undifferentiated pleomorphic sarcoma	16 (13.9%)	1 (10.0%)	9 (18.4%)	6 (10.7%)
Angiosarcoma	6 (5.2%)	1 (10.0%)	3 (6.1%)	2 (3.6%)
Endometrial stromal sarcoma	1 (0.9%)	0 (0.0%)	0 (0.0%)	1 (1.8%)
Chordoma	1 (0.9%)	0 (0.0%)	1 (2.0%)	0 (0.0%)
Dermatofibrosarcoma protuberans	2 (1.7%)	0 (0.0%)	1 (2.0%)	1 (1.8%)
Gastrointestinal stromal tumor	1 (0.9%)	1 (10.0%)	0 (0.0%)	0 (0.0%)
Rhabdomyosarcoma	3 (2.6%)	0 (0.0%)	0 (0.0%)	3 (5.4%)
Chondrosarcoma	4 (3.5%)	0 (0.0%)	0 (0.0%)	4 (7.1%)
Myxofibrosarcoma	4 (3.5%)	0 (0.0%)	1 (2.0%)	3 (5.4%)
Other bone sarcomas	11 (9.6%)	1 (10.0%)	7 (14.3%)	3 (5.4%)
Other soft tissue sarcomas	29 (25.2%)	4 (40.0%)	11 (22.4%)	14 (25.0%)
**Stage (4 vs. other)**				
Stage (0–3)	67 (58.3%)	7 (70.0%)	27 (55.1%)	33 (58.9%)
Stage 4	48 (41.7%)	3 (30.0%)	22 (44.9%)	23 (41.1%)
**NGS tumor panel completed**				
Yes	30 (26.1%)	4 (40.0%)	9 (18.4%)	17 (30.4%)

**Table 2:** Incidence of findings not predicted by clinical guidelines.

	PGV Patients (N = 10)
**Did they meet 2020 NCCN/NSGC/ACMG testing guidelines?**	
Yes	3 (30.0%)
NCCN	3 (30.0%)
NSGC/ACMG	1 (10.0%)
No	7 (70.0%)
Did they meet 2020 NCCN/NSGC/ACMG guidelines based on family history regardless of personal history? *	
Yes	3 (30.0%)
No	5 (50.0%)
Not available	2 (20.0%)

* Based on Chompret criteria.

**Table 3:** Current diagnostic testing criteria based on NCCN and Chompret criteria.

Criteria	Details
NCCN Guidelines for Classic Li–Fraumeni Syndrome Testing	Testing is recommended for individuals with: A diagnosis of sarcoma at <45 years of age A first-degree relative diagnosed with cancer at <45 years of age. An additional first- or second-degree relative in the same lineage with cancer diagnosed at <45 years or with a sarcoma at any age
Chompret Criteria (updated 2015)	Germline testing considered for individuals with: A personal history of cancer in the Li–Fraumeni spectrum (e.g., soft tissue sarcoma, osteosarcoma, CNS tumor, breast cancer, or adrenocortical carcinoma) diagnosed at <45 years, and a first- or second-degree relative diagnosed with cancer at <45 years or a sarcoma at any age. Multiple primary cancers (excluding breast) associated with Li–Fraumeni syndrome before age 46 [14]. Adrenocortical carcinoma, choroid plexus carcinoma, or rhabdomyosarcoma of embryonal anaplastic subtype at any age, regardless of family history Breast cancer before age 31 Known family history of a TP53 pathogenic germline variant (PGV)

**Table 4:** Clinical and genomic features of patients with pathogenic variants.

Age at Diagnosis	Type of Sarcoma	Family History	Pathogenic Variant	Somatic Alteration	Stage	Status	Germline Testing Eligible Per Chompret
64	Angiosarcoma	Yes (father, colon cancer at 63)	ATM (heterozygous) p.Ser160Alafs*23 NM_000051.3:c.478_482del	NA	IV	Died, 67	No
45	Epithelioid sarcoma	No	RAD50 (heterozygous) p.Leu424Glufs*7 NM_005732.3:c.1270_1271del	NA	IV	Died, 52	No
32	Gastrointestinal stromal tumor	Yes (mother, breast cancer at 57)	SDHA (heterozygous) p.Arg512* NM_004168.3:c.1534C > T	SDHA R512 TP53 N239K	II	Alive, 36	yes
33	Embryonal undifferentiated sarcoma	Yes (maternal aunt with myeloma and brain tumor at 63)	TP53 (heterozygous) p.Pro151Thr NM_000546.5:c.451C>A	NA	III	Alive, 37	
59	Pleomorphic liposarcoma	Yes (sister, breast cancer at 67)	DIS3L2 deletion Exon 9 (heterozygous) NM_152383.4:c.951-?_1124+?del	NA	IV	Alive, 62	No
42	Breast Phyllodes tumor	Yes (second-degree relatives with breast, prostate, colon, pancreatic in third and fourth decades)	NBN deletion (Exons 15–16), heterozygous NM_002485.4:c.2185-?_*2246+?del	NA	IIIA	Alive, 47	Yes
22	Osteosarcoma	Yes (mother, breast cancer at 59; brother, osteosarcoma at 26)	TP53 deletion (heterozygous) p.Arg337Cys NM_000546.5:c.1009C>T	NA	IIB	Alive, 31	Yes
59	Uterine Leiomyosarcoma	Second-degree relatives with breast cancer, colon cancer, lung cancer over age 50	TP53 deletion (promoter)Heterozygous NC_000017.10:g.7590924-?_7591611+?del	TP53 TMB 8.3 Mt/MB	IV	Died, 63	No
72	Spindle cell sarcoma	No	ATM mutation splice donor (heterozygous) NM_000051.3:c.5005+1G>A	NA	II	Died, 74	No
40	Undifferentiated pleomorphic sarcoma	NA	BRCA1 deletion (Exons 8–11) (heterozygous) NM_007294.3:c.548-?_4185+?del	NA	IIIA	Alive, 45	NA

## Data Availability

The original contributions presented in this study are included in the article. Further inquiries can be directed to the corresponding author(s).
